# Supplementary material for: Learning curve and surgical outcome of robotic assisted colorectal surgery with ERAS program
Source: Sci Rep. 2022 Nov 29;12:20566. doi: 10.1038/s41598-022-24665-w (PMC9709162; doi:10.1038/s41598-022-24665-w)
Supplement: Supplementary file 2 — Supplementary Information 2. [file 41598_2022_24665_MOESM2_ESM.zip › RAL Raw data-Ñ[▒K/076/OP N_ñw╝╨░O▒Kñσ.pdf]

W52 / W52

臺中榮民總醫院
手術室護理記錄

[redacted]

基本資料

- 室別： 開刀房-17室，手術科別： CRS，手術類別： 常規，日期： 2021/04/21
- 進入時間： 0820 ； 麻醉開始時間： 0845 ； Time Out時間： 1005 ； 劃刀時間： 1007 ； 手術結束時間： 1432 送出時間： 1515 ；
- 麻醉方式： GE
  診斷： Malignant tumor of sigmoid colon
  手術名稱： Robot Assisted Colectomy(L)
  手術局部用藥： NIL
  術前預防性抗生素：
  - 抗生素名稱一： Veterin 1GM IVD由麻醉護理師執行
  - 時間： 1000
  - 上次簽章: 周筱涵

  ，追加預防性抗生素：
- 手術醫師： 林俊余，林陳彥，陳威遠
- 麻醉醫師： VS 齊光彪
-
  - 刷手護士A ： 張秀敏，時間： 0820，刷手護士B ： 羅媗鈴，時間： 0820，接替刷手一： 王昱瑀，時間： 1200
  - 流動護士A ： 周筱涵，時間： 0820，接替流動一： 羅媗鈴，時間： 1130，接替流動二： 黃奎傑，時間： 1500

皮膚狀況

A-V Shunt： 無
術前： 完整
記錄者： 上次簽章: 周筱涵
術後： 完整
記錄者： 黃奎傑

手術準備

手術臥式 單一手術臥式

-
  - 手術臥式： 截石臥式
  - 使用附件： 矽膠枕頭，腳架，側身架，其它： 海綿
  - 皮膚準備： 10%Aq Betadine，Hibiscrub，2% Chlorhexidine
  - 電燒： 使用電燒: 電極片位置： 右大腿，記錄者： 上次簽章: 周筱涵

止血帶T： 無

植入物

- 植入物名稱/號碼/數量/部位： Ethicon CDH29A x1/S-colon
  davinci XI Endowrist stapler 45 green reload x2/S-colon

，記錄者： 上次簽章: 羅媗鈴

標本送檢

- 細菌培養 無
- 細胞學 無
- 冰凍切片 無
- 病理標本
  組織名稱一： S-colon
  ,
  組織名稱二： IMA root
  ,
  組織名稱三： distal cutend

- 截肢 無

標本交付 標本： 2 袋 ，標本： 0 盒 ，標本： 1 瓶 ，細胞學： 0 管 ，Culture Tube： 0 管 ，被交付醫師： R 陳威遠，

- 記錄者： 上次簽章: 羅媗鈴
- 時間： 1315

傷口術後數料

傷口數料： 傷口數料： 無

- 請於圖上標示部位：

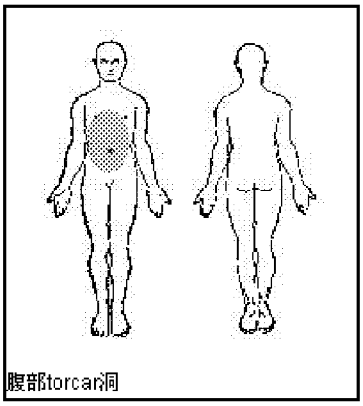

- 傷口數料： meport

導管

導管	天數	導管資訊	插入日期	插入時間	更換日期	拔除日期	拔除時間	拔除原因	預計拔除/更換	預計拔除/更換日期
Epidural PCA(1)	1	部位:硬脊膜;	2021/04/21	0850						
Foley(1)	1	號碼:18 Fr.; 部位:膀胱;	2021/04/21	0940						
Double Lumen CVC(1)	1	號碼:8 Fr.; 深度:15cm; 部位:jugular (右); 象限:RU;	2021/04/21	0930						
A-line (1)	1	號碼:20 G; 部位:左手;	2021/04/21	0945						

用物計數

- 手術中出血量： minimal c.c.
- 手術中尿量： 650 c.c.
-
    - 其它： 無

用物計數： 多切口同時縫合

- 1.器械： 正確，2.縫針： 正確，3.刀片： 正確，4.紗布： 正確，5.腹部墊： 未使用，6.紗球： 未使用，7.PEANUTS ： 未使用，8.紗條： 正確，9.絲帶： 正確，10.針頭： 未使用，11.Vessel loop： 未使用，12.Boots： 未使用，13.Jelco： 未使用，14.棉條： 未使用，
    - 第一次：
        - 刷手護士 ： 王昱琄
        - 流動護士： 上次簽章: 羅媗鈴
        - 時間： 1250

  ,
    - 第二次：
        - 刷手護士 ： 王昱琄
        - 流動護士： 上次簽章: 羅媗鈴
        - 時間： 1300

  ,
    - 第三次：
        - 刷手護士 ： 王昱琄
        - 流動護士： 上次簽章: 羅媗鈴
        - 時間： 1310

備註

備註： DAVINCI 0度SCOPE NO.1

- 記錄者： 上次簽章: 周筱涵
- 時間： 1045

填寫護理人員 黃奎傑

正式摘要

W52 / W52

doctorRank:B
